# Preconception Weight Loss to Prevent Childhood Obesity: Current Evidence and Research Gaps

**DOI:** 10.1007/s13679-025-00682-4

**Published:** 2026-02-21

**Authors:** Kimberly K. Vesco, Erin S. LeBlanc, Suzanne Phelan, Natalie A. Rosenquist, Meghan Mayhew, Janne Boone-Heinonen

**Affiliations:** 1 Kaiser Permanente Center for Health Research, 3800 N. Interstate Ave., Portland, OR 97227 United States; 2 California Polytechnic State University Center for Health Research, San Luis Obispo, CA USA; 3 Institute for Health Policy Studies, University of California, San Francisco, 490 Illinois St., San Francisco, CA 94158 USA

**Keywords:** Preconception, Pediatric obesity, Child, Weight loss, Lifestyle interventions

## Abstract

**Purpose of Review:**

The goal of this narrative review is to summarize the evidence regarding the impact of preconception weight loss and preconception weight loss interventions on child obesity, describe ongoing challenges surrounding preconception weight loss intervention research, and discuss future directions.

**Recent Findings:**

Data are sparse regarding the impact of preconception weight loss and preconception interventions on offspring outcomes beyond birth. There is insufficient evidence to determine the impact of preconception weight loss interventions on childhood obesity.

**Summary:**

Future research should include long-term observational studies and multi-center randomized trials during the preconception period among participants from diverse geographic and cultural backgrounds. Studies should be of sufficient sample size and duration to fully assess the impact of preconception interventions on childhood obesity and other important measures of child health. Ongoing challenges include those related to identification and recruitment of people prior to pregnancy, determining the optimal lifestyle intervention design to optimize impact on child health, and long duration of required follow up to examine child outcomes.

## Introduction

The prevalence of obesity has steadily increased in the United States (US) over the past several decades both for women and children. Data from the National Health and Nutrition Examination Surveys show an increase in child obesity prevalence from 10.0% in 1988–1994 to 21.1% in 2021–2023 [1, 2]. Although many early life origins of obesity are recognized [3], maternal obesity is one of the strongest and most consistent risk factors for child obesity [4]. Given that 41.3% of adult women have obesity (body mass index (BMI) ≥ 30.0 kg/m^2^), including 12.1% with severe obesity (BMI ≥ 40.0 kg/m^2^) [5], development of effective strategies to reduce the prevalence and intergenerational impacts of maternal obesity are critical but remain elusive.

National guidelines and recommendations addressing maternal obesity focus predominately on the prenatal period, including the Institute of Medicine’s 2009 gestational weight gain (GWG) guidelines and US Preventative Services Task Force (USPSTF) 2021 recommendation that pregnant persons should be offered behavioral interventions to prevent excess GWG [6, 7]. While reducing GWG is associated with lower risk of adverse pregnancy and maternal outcomes such as gestational diabetes mellitus (GDM) and large-for-gestational age delivery, pregnancy interventions do not impact risk of preterm birth or infant growth rates in the first year of life [6, 7]. Moreover, limited clinical trial data suggest that there is no significant impact of prenatal interventions on child obesity or related metabolic outcomes [8–11].

Initiating interventions in the preconception period has become a recent focus for improving both maternal and child health outcomes. The Centers for Disease Control and Prevention have identified preconception care as a primary initiative in the US [12], and clinical care guidelines from the USPSTF and the American College of Obstetricians and Gynecologists (ACOG) recommend interventions including weight loss for those with overweight or obesity in the preconception period [7, 13]. However, research on the impact of preconception weight loss interventions on child obesity is still in early stages.

The goal of this narrative review is to summarize the current evidence regarding the impact of preconception diet and lifestyle focused weight loss interventions on child obesity. We then discuss challenges and future directions for behavioral preconception weight management intervention research.

## Evidence of Effects of Weight Loss Prior to Pregnancy on Child Obesity

### Conceptual Rationale: Effects of Maternal Obesity and the in Utero Environment on Child Obesity

Mechanisms by which maternal obesity may impact childhood obesity risk include increased systemic inflammation, oxidative stress, endothelial dysfunction, epigenetic changes, hyperinsulinemia, and alterations in the maternal/infant microbiome [14–21]. Given the potential impact of these factors on the developing placenta and fetus [14–17, 22], it is postulated that preconception weight loss interventions could reduce the adverse systemic effects of obesity on fetal programming and thereby decrease risk of obesity and metabolic dysfunction in the offspring.

The Developmental Origins of Health and Disease (DOHaD) hypothesis, also known as the Barker hypothesis, proposes that the metabolic environment of the developing fetus has long-term implications for the future metabolic health of the child [23]. The original hypothesis postulated that poor nutrition and growth in early fetal life were associated with an increased risk of long-term chronic disease. Studies examining the association of small-for-gestational age at birth (SGA, birthweight < 10%ile) with longer term child and adult outcomes have shown an inverse relationship between birth weight and measures of central adiposity, insulin resistance, and metabolic syndrome [20].

Current evidence suggests that maternal overnutrition and excessive fetal growth during pregnancy (large-for-gestational age offspring [LGA], birthweight > 90%ile) are also risk factors for childhood obesity and metabolic dysfunction [20]. Animal studies have shown effects of obesogenic maternal diets on fetal programming that predisposes the offspring toward future obesity and its metabolic sequela including hyperglycemia, diabetes, and hypertension [24]. Human observational studies have shown that both excessive gestational weight gain and maternal hyperglycemia (gestational and pre-existing diabetes) are associated with risk of childhood obesity and metabolic dysfunction [17, 22, 25–28].

### Associations between Preconception Weight Loss and Maternal Obesity-related Pregnancy Outcomes that are known Risk Factors for Child Obesity

Observational data suggest that preconception weight change may impact newborn outcomes associated with intrauterine fetal growth (SGA and LGA) as well as obesity-related maternal pregnancy outcomes that are associated with metabolic dysfunction and known to impact fetal growth and child obesity risk (gestational diabetes mellitus, GDM, and hypertensive disorders of pregnancy, HDP). Most of the available data come from studies examining changes in weight between pregnancies and from longitudinal data on women who have experienced pregnancy after bariatric surgery.

Systematic reviews examining the impact of interpregnancy weight change on pregnancy outcomes have found that interpregnancy weight gain is associated with increased risk of GDM, HDP, and LGA, whereas interpregnancy weight loss is associated with reduced risk of GDM and LGA, increased risk of SGA, and no demonstrated impact on HDP [29–31]. Surgically-induced prepregnancy weight loss has been associated with reduced risk of GDM, HDP, and LGA, but may also increase the risk of poor fetal growth (SGA) [32–34]. Associations between surgically induced preconception weight loss and childhood BMI or obesity status in the offspring are mixed [35–43]. Observational data on non-surgically induced preconception weight loss and child weight-related outcomes are scant and suggest no association with child BMI, adiposity, or fat mass [44].

### Evidence of Diet and Lifestyle Focused Preconception Weight Loss Interventions on Child Weight-related Outcomes

There is an emerging body of evidence of the impact of preconception diet and lifestyle interventions on preconception weight loss and pregnancy outcomes. Several of the published trials were focused on populations with infertility and included fertility treatment following the diet and lifestyle intervention [45–47], with the primary aim to improve live birth rates. Other trials were conducted among women with overweight and obesity and have yet to report on longer-term outcomes for the offspring [48–51].

While the reporting of childhood outcomes from randomized trial data is scarce, there is little evidence that they affect fetal size at birth or maternal pregnancy-related conditions that impact long-term risk for childhood obesity. The Table 1 describes completed and ongoing trials and child outcomes reported to date. Of the published preconception diet and lifestyle intervention trials conducted outside the context of infertility, only one found an impact on early pregnancy glycemia [52] and none found a difference in the incidence of HDP or GDM. Only three trials have reported on size for gestational age, finding no effects on SGA or LGA risk, with the exception of lower risk of LGA in a trial of a modified very low energy diet (~ 800 kcal/d) with meal replacement [50]. None of these trials have yet published data regarding child BMI or growth. Some ongoing preconception diet and lifestyle interventions are specifically targeting child outcomes with planned follow up of children beyond birth (Table 1); however, it may be several years before these results are available.

**Table 1:** Completed and ongoing trials testing preconception diet and lifestyle interventions

Trial	Setting	Intervention	Primary Outcome	Child Outcomes Reported
LeBlanc et al. (Prepare) [48]	United States *N* = 326 BMI ≥ 27	Individualized phone counseling + website; weekly phone visits for 6 months then monthly until end of pregnancy.	% exceeding GWG guidelines	SGA, LGA, Birthweight z-score
Phelan et al. (GDM Prevention) [49]	United States *N* = 199 Prior GDM BMI ≥ 25	Individualized in-person and remote counseling; weekly visits for 4 months then bi-weekly until conception.	Incidence of GDM recurrence	SGA, LGA, Weight for length z-score
Price et al. [50]	Australia *N* = 164 BMI 30–55	12 week modified very-low-energy-density diet (500 kcal/day deficit) with two meal replacement shakes/day followed by quarterly visits until conception.	Maternal fasting plasma glucose at 26 to 28 weeks’ gestation.	IUGR, LGA
Rönö et al. (RADIEL) [51]	Finland *N* = 228 BMI > 30 and/or prior GDM	Nurse phone counseling every 3 months until end of pregnancy.	Incidence of GDM	Birthweight z-score
Bijlholt et al. (INTER-ACT trial) [67, 68]	Belgium *N* = 1450 People with excess GWG in prior pregnancy	E-health and face-to-face combined lifestyle intervention	Composite outcome of at least one the following: Pregnancy-induced hypertension; GDM; cesarean section; LGA	Not yet reported
Dennis et al. (HeLTI Canada) [69]	Canada *N* = 5230 People planning pregnancy within 3 years, their partners, the first child born after randomization, and, if parous, a prior child if aged 3–12 months at time of randomization	Preconception-early childhood intervention. Phone-based with public health nurses. Intervention targets child obesity risk factors: parental nutrition, physical activity, screen time and sleep	Child overweight/obesity (> 85th percentile) at age 5	Not yet reported
Erickson et al. (LIPP) [70]	USA *N* = 200 With overweight or obesity at 12 weeks postpartum	One to one nutrition counseling and supervised 60-minute physical activity sessions	Group differences in neonatal adiposity at birth assessed by PEA POD and placental mitochondrial lipid metabolism	Not yet reported
Sauder et al. (NDPP-NextGen) [71]	USA *N* = 360 With overweight or obesity	Diabetes Prevention Program video conference classes	BMI in early pregnancy (6–8 weeks gestation)	Not yet reported
Sujan et al. (Before the Beginning) [72]	Norway *N* = 167 females At risk for GDM	Exercise and time-restricted eating (≤ 10 h/day window of energy intake)	Glucose tolerance at gestational week 28	Not yet reported
Opstrup PREPARE CHILD AUH [73]	Denmark (Aarhus University Hospital) *N* = 140 couples with maternal BMI 27–44.9.9 planning pregnancy in the next 3 years (couples are recruited during pregnancy for a postpartum intervention)	Comprehensive preconception parental weight loss intervention including dietary counseling by a dietitian from 3 months postpartum of the 1 st child until birth of the 2nd child. Goal is weight loss of 10% from prepregnancy weight followed by weight maintenance until pregnancy onset.	Neonatal fat mass (child born after intervention) Difference in epigenetic changes between the child born prior to the intervention and child born after intervention	Not yet reported
Mølgaard PREPARE CHILD COPENHAGEN [74]	Denmark (University of Copenhagen) *N* = 240 females with BMI 27–44.9.9 planning pregnancy within 1 year and their partners	Comprehensive pre-conceptional parental weight loss intervention including dietary counseling and a very low-calorie diet for 8–10 weeks to introduce a rapid weight loss of approximately 10%, followed by reintroduction of foods and promotion of weight maintenance	Neonatal fat mass	Not yet reported

GWG = gestational weight gain; GDM = gestational diabetes mellitus; LGA = large-for-gestational-age; SGA = small-for-gestational age; IUGR = intrauterine growth restriction

## Challenges Faced by Preconception Interventions

The trials that have examined the impact of preconception lifestyle interventions in those planning pregnancy have faced multiple design challenges that may have limited their ability to detect an impact of the preconception intervention on pregnant people and children [53]. We list several of these challenges in the sections below.

### Identifying and Recruiting those Who are Planning Pregnancy

Surveys have shown that people considering pregnancy feel that it is important to have a healthy diet and be at a healthy weight before pregnancy and are interested in managing their weight prior to pregnancy [54]; however, identifying and recruiting women planning pregnancy is challenging. One option is to leverage electronic medical records in recruitment [55]; however, women’s pregnancy intention is not systematically documented in the medical record. Although ACOG recommends that pregnancy intention screening or assessment of reproductive life plans take place with any patient encounter, regardless of the visit reason or type of clinician seen [13], most providers do not routinely screen for pregnancy intention or provide preconception care to patients at risk for pregnancy [56]. Only about one in six obstetricians/gynecologists and family physicians provided preconception care to the majority of the women presenting for prenatal care [57]. Another approach is to identify people with diagnosis codes such as prenatal counseling or testing codes for genetic tests commonly done before pregnancy; this has been used successfully in some studies [48]; however, these are inconsistently tracked among healthcare providers and systems, and the number of people with these codes in a single health care system may not be a large enough from which to draw the needed sample size as many people do not see providers until after pregnancy onset.

### Powering the Study for the Primary Outcome Given that the Pregnancy Rate of the Recruited Population is Unknown

Estimating how many of those recruited will become pregnant and deliver babies within a grant cycle (typically 5 years) is difficult [58]. Even among those who say they are planning to get pregnant, many do not experience pregnancy in the following one- or two-year period. For example, in the Prepare trial, all women had to answer “yes” or “maybe” to the following question: “Would you like to become pregnant in the next 2 years?” Yet only 52% experienced a singleton pregnancy during the study timeline [48]. Moreover, the timing of pregnancy varied; 33% became pregnant in the first 6 months of enrollment, 51% between 6 and 24 months, and 16% after 24 months [48].

Power calculations require an accurate prediction for how many of those joining the study will experience pregnancy and give birth during the study timeline. The rates of pregnancy in preconception lifestyle trials have ranged from 33 to 62%. In other words, 38 to 67% of the participants who received the preconception intervention were not included in the primary outcome analyses [48–51]. Given that the outcomes of most interest for childhood obesity such as maternal GDM and neonatal LGA and SGA are relatively uncommon (< 30%), even in a population with obesity, preconception studies require large sample sizes to be adequately powered to collect meaningful data on child outcomes. One way to potentially decrease the need for such a large sample is to focus on those at highest risk of pregnancy-related complications that impact childhood growth such as those with previous LGA or GDM; however, this further limits the population pool as well as the generalizability.

### Determining Ideal Intervention Intensity and Modality

In the preconception weight loss trials to date, mean weight loss has been relatively modest. Maximizing weight loss while ensuring the intervention is safe and doesn’t result in rebound weight gain in pregnancy are important considerations. Given the known increased risk of weight regain in nonpregnant adults after an intensive weight-loss phase [59–61], it is critical to include and administer ongoing intervention with frequent contact to prevent weight regain before, during, and after pregnancy [62]. Maintaining participant engagement long-term is challenging, and also frequent intervention contact for a long duration can be cost-prohibitive. Adaptive interventions that can adjust intervention intensity based on participants’ weight and lifestyle status could be studied for preconception interventions given the variability in timing of pregnancy.

### It Takes Many Years of Follow-up to Examine the Impact of Pre-pregnancy Weight Loss on Childhood Growth

In order to study the impact of a preconception lifestyle intervention on childhood obesity risk, a study would require many years of intervention and follow-up. Beyond requiring substantial resources, long-term follow-up studies often have reduced participation over time, particularly among populations with more adverse social determinants of health.

### Trials of those with Infertility

Studying preconception interventions in those with infertility can lower some of the barriers discussed above because it is easier to predict the percentage of participants who will likely get pregnant and the timing of pregnancy. However, trials of lifestyle interventions in people with infertility are only applicable to a small proportion of the pregnant population (approximately 15%) [63], and the fertility medications and treatments themselves can impact pregnancy and neonatal outcomes making it difficult to disentangle intervention effects from infertility treatment effects [64–66].

### Charting the way Forward: Future Research Needs

**Fig. 1:**
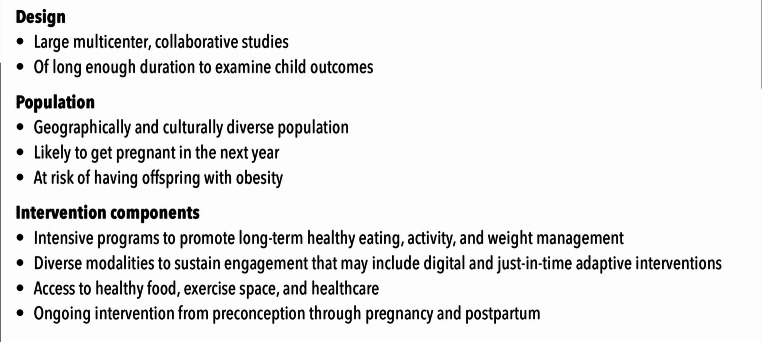
Key study elements to assess the impact of prepregnancy weight loss on childhood obesity

There are several key elements to consider when designing and testing interventions seeking to reduce childhood obesity through preconception weight loss (Figure 1). Given the challenges facing research seeking to examine the impact of preconception weight loss interventions on childhood obesity, study designs such as multi-center randomized clinical trials and other collaborative designs are needed. Studies should include diverse populations and algorithms and strategies to identify populations who are most likely to experience pregnancy in the next year and who are at high risk of having offspring with obesity. Testing novel digital health modalities and family-based interventions as well as reaching communities at highest risk for pregnancy-related morbidity and mortality and later chronic disease should be high priorities.

## Conclusions

Despite being recommended by several organizations, the impact of maternal lifestyle interventions before pregnancy on offspring childhood obesity is unclear. Innovative long-term studies that integrate pediatric and maternal health perspectives in diverse settings and backgrounds are needed to fully elucidate the link between preconception maternal weight and childhood obesity risk.

## Data Availability

No datasets were generated or analysed during the current study.

## Key References

- Hu K, Staiano AE. Trends in Obesity Prevalence Among Children and Adolescents Aged 2 to 19 Years in the US From 2011 to 2020. *JAMA Pediatr.* 2022;176(10):1037–1039.
  - Childhood obesity has been increasing among children 2–19 years of age, with a significant increase among the subgroup of those aged 2–5 years.

- Creanga AA, Catalano PM, Bateman BT. Obesity in Pregnancy. *N Engl J Med.* 2022;387(3):248–259.
  - Maternal obesity in pregnancy is associated an increased risk of adverse maternal, fetal, neonatal and infant outcomes.

- Seneviratne SN, Rajindrajith S. Fetal Programming of Obesity and Type 2 Diabetes. *World J Diabetes.* 2022;13(7):482–497.
  - In utero exposure to factors associated with maternal obesity, such as an abnormal metabolic milieu, increase the risk of obesity and type 2 diabetes in the offspring.

- Cristian A, Tarry-Adkins JL, Aiken CE. The Uterine Environment and Childhood Obesity Risk: Mechanisms and Predictions. Curr Nutr Rep. 2023;12(3):416–425.
  - Suboptimal intrauterine environments program fetal health and interact with the postnatal environment and maternal and fetal genetics to contribute to the risk of childhood obesity.

- Wing RR, Caughey AB, Phelan S. Preconception Weight Loss to Improve Pregnancy. Outcomes: Does the Evidence Justify National Recommendations? *Obesity (Silver Spring).* 2023;31(3):594–596.
  - The evidence base on the effects of preconception weight loss intervention on pregnancy outcomes is limited.

- Yu Y, Lyo V, Groth SW. The Impact of Maternal Bariatric Surgery on Long-term Health of Offspring: A Scoping Review. *Pediatr Res.* 2023;94(5):1619–1630.
  - Maternal bariatric surgery impacts offspring health, including effects on weight status and obesity, but results are mixed in terms of direction of association.
